# Editorial: Ethical considerations of large language models: challenges and best practices

**DOI:** 10.3389/fdgth.2026.1807664

**Published:** 2026-03-09

**Authors:** Pedro Elkind Velmovitsky, Luk Arbuckle, Paraskevi Papadopoulou

**Affiliations:** 1 Centre for Digital Therapeutics, University Health Network (UHN), Toronto, ON, Canada; 2 Institute of Health Policy, Management and Evaluation, University of Toronto, Toronto, ON, Canada; 3 IQVIA Applied AI Science, Ottawa, ON, Canada; 4 American College of Greece, Athens, Greece

**Keywords:** access, artificial intelligence, bias, equity, ethics, governance, large langauge models

The rapid adoption of large language models (LLMs) presents transformative opportunities alongside profound ethical challenges (1). LLMs are reshaping how health systems learn, decide, and communicate. This Research Topic distills ten contributions into five practice-oriented themes: governance and accountability; equity and access; privacy and defensibility; method rigor and evaluation; and right-sized deployment. These themes do not exist in isolation but form a complex web of interdependencies that should be addressed holistically.

## Governance and accountability

Responsibility for LLM-assisted decisions leading to adverse outcomes remains unresolved, representing a critical gap in terms of wide-scale adoption. Fareed et al.'s systematic review highlights gaps in accountability treatment, proposing regulatory safeguards, technical controls, human oversight, and transparency/accountability are necessary for clinical integration. Qi and Pan extend this by examining general-purpose LLMs in evidence-based medicine tasks, highlighting risks including disembodiment (separation from clinical context), deinstitutionalization (bypass of review processes), and depragmatization (loss of clinical judgment). LLMs may also exhibit other limitations including numeric errors and unverifiable citations – reinforcing the need for auditable, reviewable workflows (2).

LLMs can greatly increase access in resource-constrained environments. A developing-nation perspective on medical education shows that LLMs can improve access and learning but introduce risks including plagiarism and misinformation. This underscores the need for clear AI use policies, authorship rules, and training for faculty and students (Jaleel et al.). Tung et al.'s survey further confirms that fragmented governance requires multi-layered socio-technical frameworks integrating technical fixes with robust oversight and legal guidelines.

## Equity and access

Bias and fairness concerns appear in 26% of studies examined by Fareed et al., while Chan and Kwek demonstrate that LLMs assigned higher cardiovascular risk to men and Black or South Asian patients. Notably, race-based decisions remained stable across contexts while sex-based judgments varied, suggesting deeply embedded biases (Fareed et al.). This study also revealed inconsistent citations, hallucinations, and systematic omission of social determinants of health as related risks, echoing broader evidence that systems trained on historical data can perpetuate existing health disparities (3).

At the infrastructure layer, biobanking-related work focuses on *size, site, access,* and *speed:* prioritizing quality-over-volume, recognizing biobanks as socio-technical “boundary objects,” coupling FAIR with fairness and data sovereignty, and maintaining human oversight as AI accelerates workflows (Mayrhofer). While data networks build critical mass and increase adoption, scale carries strategic and political power that can exacerbate inequities without careful governance. Jaleel et al. emphasize that the digital divide in developing nations creates unequal LLM access (4).

## Privacy and defensibility

Privacy vulnerabilities demand technical and governance solutions. DP-CARE 's framework performs differentially private, classifier-only training atop a frozen domain encoder, formally bounding privacy loss while favoring recall where missed positives are costlier, at a modest compute overhead – demonstrating the feasibility of privacy-preserving training in sensitive mental health applications (Karpontinis and Soufleri). The mathematical foundations of differential privacy, which bound the influence of any individual training record, provide the formal guarantee underlying such approaches (5). Mayrhofer complements this with infrastructure-level privacy frameworks balancing AI advancement with data sovereignty. Tung et al. identify privacy as one of four major risks requiring multi-layered technical, procedural, and security solutions.

## Method rigor and evaluation

Several studies tested different LLMs in a number of use cases and conditions. Nantakeeratipat used an ambiguity-probe audit (structured clinical vignettes with clear-cut and intentionally ambiguous cases) to show that apparent errors come from different failures modes, distinguishing *bias* from *diagnostic boundary instabilit*y – a crucial difference for mitigation strategies (dataset diversification vs. edge-case calibration). Models exhibit “model-specific ethical fingerprints”, requiring ambiguity-sensitive evaluation and periodic re-audits.

Complementing this, HEAL-Summ illustrates multi-dimensional evaluation for health communication summarization, evaluating outputs across semantic consistency, readability, lexical diversity, emotional alignment and toxicity. Notably, this evaluation approach is paired with low-resource deployment, supporting scalable health communication while flagging different kinds of potential harms (Fisher et al.). A comprehensive bias taxonomy such as the one described in Mehrabi et al. provides conceptual grounding for such multi-dimensional evaluation efforts (6).

## Right-sized deployment

Matching model capabilities to contexts and resources emerges as critical. Fisher et al. demonstrate that smaller, specialized models provide effective, ethical communication without large-scale infrastructure. A companion engineering-focused review underscores that automation gains validation, standards, and human-in-the-loop safeguards for safety-critical contexts (Nguyen and Kittur). Jaleel et al. argue that benefits in resource-constrained environments must be balanced against integrity risks. The fact that deployment context, not capability alone, determines whether AI safely serves the population of interest is reinforced in literature, such as in Rajpurkar et al.'s perspective on AI in health and medicine (7).

## Critical insights and future directions

Informed by the papers mentioned above, three key insights emerge: first, Nantakeeratipat's distinction between diagnostic instability and bias suggests a multi-methods approach is essential to resolve inaccuracies in LLM deployment. For example, bias requires dataset diversification while instability requires improved training on edge cases. Second, governance and accountability mechanisms are necessary to ensure the success of LLM-based interventions; Karpontinis and Soufleri's differential privacy requires complementary governance, while Nantakeeratipat's methodology requires institutional commitment. Third, solutions must account for global inequities. Fisher et al. and Jaleel et al. show that more traditional approaches may prove inappropriate for resource-limited settings.

LLMs are now present across health and education. Used well, they can improve access, quality, and efficiency; used poorly, they can amplify inequities and erode trust. The contributions here point to a practical charter for implementation: institutionalize governance and auditability; embed equity, fairness and integrity; perform multi-method evaluation beyond accuracy; design for privacy and defensibility; and implement right-sized deployment with experts-in-the-loop. We hope these insights support policy, pedagogy, and system design that promote, rather than undermine, health equity, patient safety, and clinical excellence.
